# Seeing destinations through short-form videos: Implications for leveraging audience involvement to increase travel intention

**DOI:** 10.3389/fpsyg.2022.1024286

**Published:** 2022-10-14

**Authors:** Jiayu Han, Gege Zhang, Shaogui Xu, Rob Law, Mu Zhang

**Affiliations:** ^1^Shenzhen Tourism College, Jinan University, Shenzhen, China; ^2^School of Management, Jinan University, Guangzhou, China; ^3^Asia-Pacific Academy of Economics and Management, University of Macau, Macau, China; ^4^Department of Integrated Resort and Tourism Management, Faculty of Business Administration, University of Macau, Macau, China

**Keywords:** short-form travel videos, audience involvement, travel intention, destination image, psychological distance

## Abstract

Short-form travel videos are popular, but the process of audience involvement while watching remains unclear. This study explores audience involvement along with expressions of travel intention and introduces the concepts of destination image and psychological distance to construct a structural model. A total of 487 questionnaires were used for structural equation model testing. Results show that audience involvement has a positive impact on the destination’s cognitive and affective image, ultimately leading to travel intention. Meanwhile, the destination’s cognitive and affective image play a partial mediating role between the influencing mechanisms. Moreover, psychological distance has a negative moderating effect between audience involvement and travel intention, and on audience involvement and cognitive image. However, it has no significant moderating effect on both audience involvement and affective image. The results provide a broader research perspective for the development of short-form travel videos and provide important implications for destination marketing.

## Introduction

With technology’s continuous development, many destinations and attractions are now using new marketing tools to market their destination by combining them with the latest technologies. Short-form travel video, one of these new and popular marketing tools being used (Cheng et al., 2020). By documenting their own travel experiences through short videos of 15–60 s, travelers can present the food, drink, accommodation, and scenery of a destination to audiences in both real-time and real life, quickly capturing the attention of potential tourists (Du et al., 2020). Compared to pictures and text, positive short-form travel videos can make viewers feel more immersed in the local destination. Simultaneously, as tourists’ perceptions of tourism products and destinations greatly depend on the effectiveness of the information conveyed, short-form travel videos break spatio-temporal constraints while vividly showing local tourism attractions and presenting the local customs and human characteristics in a more intuitive way (Du et al., 2020). The creators share their travel experiences through the camera, exchanging travel tips and destination location information with the platform’s audience. Compared to traditional marketing methods, short videos present a stronger sense of engagement and can infect audiences with immersive and interactive experiences and provide an immersive involvement (Xu et al., 2020; Zhang, 2020). Audiences of short videos can also interact in the comments section to review the video and learn about the destination. The interaction in the comments has the opportunity to become premium experiential content and also has the advantage of trust in third-party recommendations, which can enhance the audience’s understanding of the destination and increase the likelihood of a trip. These videos have therefore become popular tourism marketing tools for tourism destinations in today’s “Internet era” and have thus received considerable attention from researchers.

Despite the boom in the literature related to short-form travel videos, this research nonetheless identifies several research gaps by reviewing past studies. Firstly, short-form travel videos mainly involve two types of tourists: creators and non-creators, with most articles exploring perspectives from the former. For example, sharing posts on travel-related social media categories by using hashtags such as “#wonderfuljourney” and storing meaningful travel experiences in a “public” diary are reasons why tourists engage with short-form video sharing platforms (Du et al., 2020). Hedonism and altruistic motives have the greatest effect on travel video creators (Kang and Schuett, 2013; Munar and Jacobsen, 2014). Articles exploring audiences’ perspectives also remain few. Secondly, various articles explore the relationship between short-form videos and other variables such as travel intention and tourist engagement behaviors (Cao et al., 2021; Chi et al., 2022), but most focus on direct relationships and less on the underlying influencing mechanisms. Furthermore, the majority of articles in this stream of literature explore the developed audience responses while in direct contact with the destination. Less attention, however, is given to the responses generated through the viewing of these short-form travel videos. Thus, the process of creating an emotional connection with the destination through short-form videos is essentially overlooked.

To broaden the scope of research in this field, this study finds that short tourism videos can not only fully reflect the unique personality of a destination, but also build a positive image of the destination and tourism brand. Therefore, how to attract tourists through short videos and other means, and attract the attention and interest of potential travelers with quality content and scenario-based travel experiences, has become an urgent issue for tourism development. Hence, to provide better insights into short-form videos in the travel context, this study takes involvement theory and media theory as the theoretical basis, and seeks to answer three research questions: (1) How does audience involvement influence travelers’ willingness to travel in the context of short travel videos? (2) What mediating role do cognitive and affective images play in this relationship? (3) How does psychological distance moderate and influence the relationship? This study enriches the relevant research in the field of tourism short-form video marketing and will provide practical implications and guidance for destination marketers on how to use short-form video marketing to deliver more experiential tourism messages and create more opportunities to interact with potential tourists. The results of this study will also provide some insights for local managers and marketing organizations to improve the effectiveness of tourism short-form video communication.

## Literature review

### Audience involvement

Audience involvement is regarded as a complicated concept in the field of communications. Previous studies posit audience involvement as parasocial interaction which is an interactive form linking program roles and audiences (Hartmann, 2016). Currently, scholars have argued that audience involvement is a multidimensional concept, using both the cognitive, affective, and behavioral aspects of parasocial interaction to measure audience involvement along with referential reflection in the final assessment. Audiences engage in reflexive and supra-social interactions with certain media programs, leading to cognitive and behavioral changes (Sood, 2002). Referential reflection is another important component of audience involvement and refers to the extent to which audiences pay attention to media messages and incorporate them into their own lives. Furthermore, Kim (2012) based on previous research, proposed the three dimensions of behavioral involvement, emotional involvement, and referential reflection to measure audience involvement when watching a program. Fu et al. (2016) likewise used these three dimensions when exploring audience involvement in reality travel shows. Therefore, following existing literature, this study defines audience involvement as the supra-social interaction and reflection generated by the audience through the tourism information of the destination presented in short-form videos. It follows Kim’s three dimensions of emotional involvement, behavioral involvement, and referential reflection to measure audience involvement in the process of watching these videos (Kim, 2012).

### Destination image

Destination image has long been a topic of widespread interest among researchers in the discipline as an important tool in tourism marketing. The concept represents beliefs, perceptions, and impressions that individuals have about a destination in sum (Crompton, 1977), and is defined as an individual’s attitude towards the attributes of a destination based on their knowledge and feelings (Moutinho, 1987; Chew and Jahari, 2014). Specifically, the destination image is formed from an objective assessment and is a collection of cognitions and emotions (Mazursky and Jacoby, 1986; Zhang et al., 2014). The ‘Cognitive-affective’ model proposed by Baloglu and McCleary (1999) suggests that destination images consist of both cognitive and emotional images. Cognitive images focus on the assessment of objective attributes of a place and relate to attitudes and knowledge of the destination, while affective images focus on sensory or emotional responses to features of the place and environment and relate to emotions such as excitement, pleasure, arousal, and relaxation (Xu et al., 2018; Binh and Bagul, 2020). The model is now widely recognized by scholars and is currently the most widely used construct. Most studies have concluded that cognition and emotion have a hierarchical relationship in assessing destination image and visitor decision-making processes (Gartner, 1994). As the ‘Cognitive-affective’ image has a high degree of maturity in empirical research and can provide a solid and detailed reference value for this study, this study will focus on the construction of a two-dimensional structure of the ‘cognitive-emotional’ image of tourism destinations by referring to this model.

### Travel intention

Travel intention is seen as a subset of tourists’ behavioral intentions. It is mainly used to describe a potential traveler and represents the traveler’s plans or willingness to visit a future destination (An et al., 2021). Woodside and Lysonski (1989) suggest that travel intention is a tourist’s likelihood of potentially traveling to a destination. Some scholars have also argued that travel intention is the likelihood that an individual will travel to a destination influenced by internal and external information such as motivation, awareness, attitudes, and other psychological dispositions (Wang, 2012). Previous studies show that the intention to travel or the decision to visit a destination is a complex process (Davari and Jang, 2021), and is influenced by external factors such as destination image (Tan and Wu, 2016), word of mouth (Tapanainen et al., 2021), and distance (Kah et al., 2016), and internal factors such as tourists’ attitude (Wang et al., 2022) and tourists’ psychology (Zhang et al., 2021). Integrating existing studies, this paper argues that travel intention is the tendency of potential tourists to visit a particular destination triggered by both internal and external information.

### Psychological distance

Psychological Distance is derived from the Construal Level Theory (CLT) in social psychology. CLT suggests that people’s interpretations of objects change systematically with their perception of psychological distance, affecting individual decision-making behavior. Psychological distance is the actor’s perception of the probability of the described event or behavior occurring in both spatial and temporal proximity while using the self as the reference point (Fernández-Ruano et al., 2022). In recent years, scholars have focused on the moderating role of psychological distance in individual judgments and behavioral decisions, and have empirically tested psychological distance as a moderating variable. Wang et al. (2020) proposed that psychological distance plays a moderating role between eco-friendly products and purchase intention. In the field of tourism, Frías-Jamilena et al. (2022) found that psychological distance moderated the relationship between gamified experiences and pro-environmental knowledge, attitudes, and behaviors. The influence of the gamified environmental interpretation on pro-environmental knowledge, attitudes, and behaviors will be significantly greater when individuals exhibit greater psychological distance. Therefore, this study draws on the concept and dimensions of psychological distance proposed by Fernández-Ruano et al. (2022) and likewise argues that psychological distance refers to the cognitive separation between tourists themselves and elements depicted in the short-form travel videos (e.g., person, events, or times), and contains temporal, spatial, and social distances. Even if the same destination information is conveyed to different individuals, people will perceive differences in psychological distance due to their own experiences, which will affect their actions after interpreting the information. When tourists believe that the destination is far away in space, it takes a lot of time to arrive, and there are many uncertain factors in the destination, the psychological distance will be correspondingly farther. Psychological distance may affect the relationship between audience involvement and travel intention. Therefore, this study introduces psychological distance into the field of short-form tourism video to explore its moderating effect on this relationship.

### Medium theory

Originally proposed by Meytowitiz, the Medium theory is an important contribution to explaining the development of new media technologies and their social impact (Meyrowitz, 1995). It encompasses the concept of audience in context and emphasizes the importance of audience in media and social relations. At the same time, it affirms the role of the media, arguing that the introduction and widespread use of new media constructs diverse contexts that may lead to changes in social behavior. With the continuous development of internet technology and digital information, audiences are more inclined to access more intensive and quality information more simply and easily, and the short-form videos fit the aforementioned user needs. In addition, it has also changed people’s social behavior and the sources of access to information. Nowadays, the development of social media is increasingly diversified, from traditional radio and television to digital mobile networks, which is changing the way of communication and people’s social behavior. Currently, short-form videos are becoming one of the main media for information dissemination, as they are deeply integrated with 5G, artificial intelligence, and other new-generation information technologies. The “media-context-behavior” framework proposed by the Medium theory fully reveals the new social relationship and behavioral changes brought about by the new digital media, which will provide theoretical and framework guidance for this paper to study the influence of tourism short-form videos on potential tourists’ behavioral intentions.

### Involvement theory

Involvement theory has its roots in social psychology and was first used to study the attitudes of individuals towards social events. Involvement is considered to be an internal psychological state that is influenced by an individual’s internal needs, values, and interests (Zaichkowsky, 1985). As involvement mediates the audience’s response to short-form video marketing, it is influenced to a certain extent by their own needs, environment, and information sources. The higher the perceived importance of the self, the higher the attention an individual will assign to it and the deeper the involvement, leading to changes in perception and behavior. In the field of tourism, tourism involvement has been well researched and reflects the internal psychological state of a tourist’s participation in tourism activity, such as satisfaction, pleasure, and self-affirmation (Wang and Li, 2018). Involvement in this study refers to the supra-social interactions and reflexes generated by audiences through the tourism experiences or destination tourism information presented in short videos in the media context, which further leads to cognitive and behavioral changes. It focuses on indirect involvement with the destination through the media platform of short-form videos.

## Hypotheses

### Audience involvement and travel intention

When watching short-form videos, audiences will link the content with their personal experiences. Similar travel experiences will have a greater impact on travel intentions and itinerary development (Munar and Jacobsen, 2014). Moreover, the perceived authenticity of the audience may cause subsequent reflections, leading them to identify with the characters or the content, influencing the audience’s attitude toward the place where the short video was originally filmed (Brown, 2015). Audiences can interact with short-form video publishers by liking, commenting, and sharing, and even possibly perceiving media characters as their close friends (Kim, 2012). The audience’s affection for the media persona may also outwardly transfer to the filming location, ultimately being attracted to the scenery and destination (Li et al., 2015). Scholars have recently found a positive relationship between audience involvement in media programs and tourists’ willingness to travel, with a higher level of audience involvement translating to a stronger willingness to visit the destination. And among the three dimensions of audience involvement, behavioral involvement, emotional involvement, and referential reflection have a positive effect on travel intention (Long et al., 2021). As a popular social media tool, short-form travel videos play important roles in audiences’ choice considerations and travel decisions (Liu and Yan, 2021). In a regular travel scenario, audience involvement may arouse the interest of potential tourists in the destinations shown in the short videos, forming their intention to travel. Hence, this study proposes the following research hypothesis on the relationship between audience involvement and travel intention.

*H1*: Audience involvement has a significant positive effect on travel intention.

### Audience involvement and destination image

Watching media programs enhances the audience’s perception of the destination’s image—the more often and the more attentive the viewer is, the better their perception is of the destination image (Muhoho-Minni and Lubbe, 2017). These positive videos incorporate symbolic carriers such as attractions, food, and folklore into the content, presenting tourism elements of the destination and forming a series of destination symbols. The audience receives these destination symbols, generates their assessments and judgments based on this narrative content, and ultimately forms a cognitive image of the destination. Influenced by publishers’ recommendations and positive emotions, audiences may also resonate with their travel experiences and thus have a favorable impression of the destination image (Cheng et al., 2020). More engaged audiences are more likely to seek out other media content related to the destination and develop a perception of the destination image by repeatedly viewing similar travel information. Furthermore, audiences gain more information about the destination by browsing related websites and participating in social interactions, forming a more colorful image of the destination. Therefore, it is posited that audience involvement has a positive impact on destination image. Some scholars argue that destination image is composed of both cognitive and affective components, verifying the significant positive impact of audience involvement on destination image (Fu et al., 2016). This study again proposes a hypothesis on the relationship between audience involvement and cognitive and affective image based on the overall two-dimensional concept that the destination image is composed of both basic cognitive and affective images.

*H2a*: Audience involvement has a significant positive effect on cognitive image.

*H2b*: Audience involvement has a significant positive effect on affective image.

### Destination image and travel intention

Destination image stems from tourists’ assessment of the elements and attributes of a destination (Park et al., 2017). Because tourists are unable to personally experience products and services prior to visiting, destination image becomes particularly important when making travel decisions. The key role of destination image in the formation of tourism decisions and travel intentions has attracted much scholarly attention. Previous studies have repeatedly verified the correlation between destination image and willingness to travel (Chen and Tsai, 2007; Bigné Alcañiz et al., 2009). The cognitive image of a destination has a significant positive effect on tourists’ behavioral intentions. Viewers are immersed in stories and scenarios while watching media programs which form their perceptions and understanding of destination images and contribute to tourists’ behavioral intentions (Fu et al., 2016). The same results were found by Hongwei et al. (2017) by exploring the impact of destination images on tourists’ behavioral intentions based on affective evaluation theory. Creating a positive, upbeat image of the destination is key to attracting visitors. If tourists have a more positive impression of a destination, they are more inclined to choose that destination when they are planning (Quintal and Phau, 2015).

By defining destination image as a multidimensional structural variable for research, a more in-depth and comprehensive understanding of the impact of destination image on tourists’ intention to travel can be obtained. The two-dimensional structure of ‘cognitive-affective’ has been widely used in destination image research leading scholars to conclude that “cognition influences emotion” (Baloglu and McCleary, 1999). This study introduces the ‘cognitive-affective’ two-dimensional structure into the model and argues that the audience’s cognitive image of the destination influences the affective image. Hence, this study proposes another hypothesis on the relationship between cognitive image, affective image, and travel intention.

*H3*: Cognitive image significantly influences affective image.

*H4a*: Cognitive image has a significant positive effect on travel intention.

*H4b*: Affective image has a significant positive effect on travel intention.

### The mediating role of destination image

Destination image is the antecedent of tourists’ behavioral intentions and the starting point for determining tourism decisions, the formation of which is also influenced by other factors. The mediating role of destination image between other variables and tourists’ behavioral intentions has been extensively validated in previous studies, such as those focusing on emotional experience (Prayag et al., 2017), perceived risk (Chew and Jahari, 2014), perceived value (Moon et al., 2013), service quality (Loi et al., 2017), and perceived constraints(Chen et al., 2013). The higher the level of involvement in the destination, the more conducive it is to form a good image of the destination and trigger more positive behavioral intentions. As an important source of information for tourists, social media tools such as short-form videos can influence their perception of destination image, which in turn plays a key role in travel intentions and travel decisions. The full mediating role of destination image between social media use and intention to travel has been verified (Huang et al., 2018). Audiences who have positive feedback on stories or images from social media programs likelier create better destination images and thus have a higher probability of traveling to the actual destination (Peralta, 2019). Existing research has confirmed the mediating role of destination image between audience involvement and travel intention. Summarily, this study proposes the hypothesis that destination images mediate the relationship between audience involvement and travel intention.

*H5a*: Cognitive image plays a mediating role between audience involvement and travel intention.

*H5b*: Affective image plays a mediating role between audience involvement and travel intention.

*H5c*: Cognitive-affective image plays a mediating role between audience involvement and travel intention.

### The moderating role of psychological distance

Even when travel videos convey the same destination information to audiences, people may perceive psychological proximity or distance from the destination differently due to their own experiences, influencing their actions in response to the interpretation of the travel information. The perceived stress of traveling is more pronounced and costlier if people perceive the distance between the destination and their place of residence to be longer (Xu and Li, 2018). Longer psychological distance brings more information and risk uncertainty, thus lowering the image of the destination even if the audience’s higher level of involvement with short travel videos is offset to some extent (Day and Bartels, 2008; Darke et al., 2016). Furthermore, the psychological distance also affects the relationship between audience involvement and travel intention and deters the travel decisions of potential tourists. For a particular destination, a longer psychological distance is expected when the traveler perceives the destination to be spatially distant, takes a significant amount of time to reach, and when the uncertainty of the destination is high. Here, even if the audience generates good reflexes and very social interactions after watching the travel-based short videos, the likelihood of actual visits will nonetheless be affected when the psychological distance is far. Hence, also two other hypotheses are proposed by this study.

*H6*: Psychological distance negatively moderates the relationship between audience involvement and travel intention.

*H7a*: Psychological distance negatively moderates the relationship between audience involvement and cognitive image.

*H7b*: Psychological distance negatively moderates the relationship between audience involvement and affective image.

**Figure 1:**
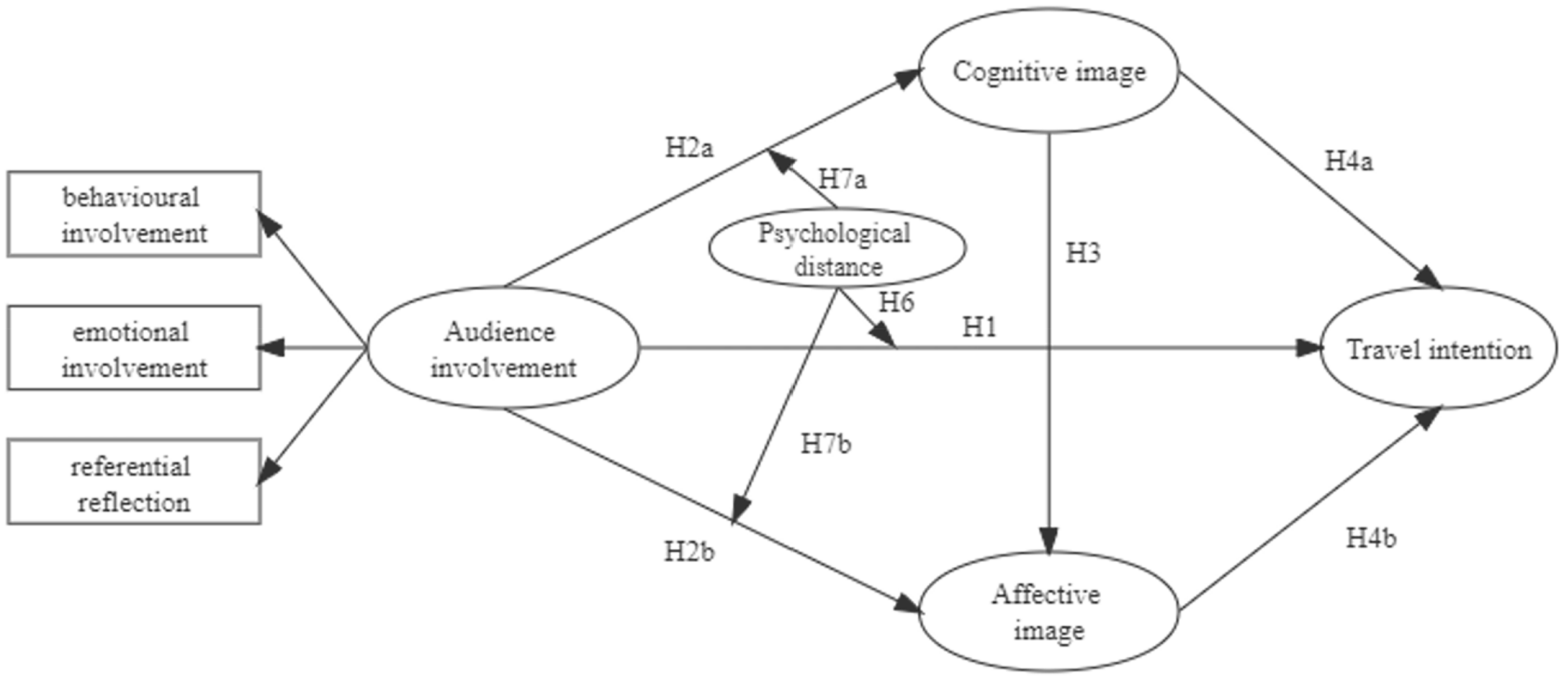
Theoretical model and hypotheses.

Based on the abovementioned assumptions, a structural equation model was constructed as shown in Figure 1 below:

## Methodology

### Survey instrument

The main body of the survey questionnaire is divided into two parts. The first part is the demographic survey, which is mainly used to collect basic information of respondents, including gender, age, education, income, and occupation. In addition, the researchers also investigated the number of tourists’ annual trips, the frequency and length of short-form videos viewed in their daily life, and whether they had any experience of visiting a destination after watching a short-form video about a destination. The second part is the core content of the questionnaire, which mainly measures the real feelings of respondents when watching short-form tourism videos, including audience involvement measurement, destination image measurement, psychological distance measurement and travel intention measurement. This part is compiled with the Likert five-point scale.

The Audience Involvement Scale (AIS) for this study was based on the AIS developed by Kim (2012) and revised by Fu et al. (2016). The questionnaire consisted of 14 questions and was divided into three dimensions: behavioral involvement, emotional involvement, and referential reflection. Regarding the destination image measure, this study drew from the cognitive-emotional structure of destination image proposed by Baloglu and McCleary (1999). Meanwhile, the Cognitive Image drawn from the scale developed by Baloglu and McCleary (1999) and revised by Chew and Jahari (2014), which has 13 items to measure the cognitive image of a destination on three dimensions: quality of experience, attraction, and value. The Affective Image Scale draws on a 5-point scale of variance containing four sets of emotional adjectives developed by Russell and Pratt (1980). Second, the measure of psychological distance draws on the research scale developed by Liberman and Trope (1998), Bar-Anan et al. (2006) and adapted by Xu and Li (2018). Finally, the travel intention scale developed by Baker and Crompton (2000) and adapted by Luo and Ye (2020) was used, with specific questions modified to suit the actual study context.

The pretest was conducted from 25 June to 30 June 2022 to confirm the clarity, reliability, and comprehensiveness of the questionnaire. During this period, the author distributed a total of 153 online questionnaires through the Questionnaire Star platform.^1^ Ultimately, 141 valid questionnaires were returned, with a valid return rate of 92%, and the data from these 141 samples were analyzed in a pretest. The reliability test results showed that Cronbach’s Alpha coefficient of the total scale was 0.912, and the reliability coefficient exceeded 0.9, indicating that the overall scale was highly reliable. At the same time, Cronbach’s Alpha coefficient of each observed variable is greater than 0.7, which indicates that the variable has good internal consistency reliability. In terms of the validity test, the results of KMO and Bartlett’s sphericity test showed that the KMO value of the total scale was 0.872, indicating that the overall validity of the questionnaire was good. The KMO values of the subscales were all greater than 0.7, and Bartlett’s sphericity test value was significant (Sig. < 0.001), indicating that the validity of the questionnaire met the premise requirements of factor analysis. In addition, The researcher further adjusted and revised the questionnaire for any ambiguity in the expressions and inaccuracies in the questions considering the suggestions from the respondents, tutors, and students during the pre-test. And based on the feedback from the pilot test, one travel intention item was removed from the questionnaire because it was redundant and not clear enough.

### Data collection and data analysis

This study was conducted in China from 3 July to 15 July 2022. The convenience sampling method was used to distribute the questionnaire, because it was easier to conduct in the context of the COVID-19 pandemic. The respondents were users who had watched positive short-form travel videos using mobile phones, tablets, and other mobile devices. The questionnaires were collected mainly through online channels, mainly by re-posting the electronic questionnaires produced by Questionnaire Star on China’s most popular social media platforms such as WeChat, Xiaohongshu, and Weibo(According to statistics, as of 30 June 2022, WeChat had 1.299 billion monthly active users, Weibo was 582 million and Xiaohongshu was 200 million. The social media chosen for this study have a large influence on the marketing market). A total of 522 questionnaires were distributed online, and invalid questionnaires were identified according to the following criteria: (1) whether the questionnaire was complete; (2) whether there were multiple consecutive questions with the same choice; (3) whether the questionnaires that took less than 50s to complete were deleted. A total of 35 invalid questionnaires were excluded and 487 valid questionnaires were collected with an effective rate of 93.3%.

Further, this study analyzed the collected data through statistical software. First, SPSS 23.0 was used to conduct demographic difference analysis, descriptive statistics analysis of samples, reliability test, exploratory factor analysis, and others. Second, Amos 23.0 was used to construct structural equation models, conduct Confirmatory Factor Analysis (CFA), path coefficient analysis and mediating effect test, and others, with SPSS23.0 used to test the moderating effect of psychological distance. Finally, this study provides subsequent analysis and discussion based on the results of the abovementioned data.

## Results

### Characteristics of respondents and samples

Following Table 1, 257 (52.88%) respondents were male while 230 (47.2%) were female and the proportions of both were almost the same. Most respondents were aged 26–35 (33.3%), followed by those who were 18–25 (32%). On educational background, undergraduate students accounted for 36.3% of the population, those with college education accounted for 25.1%, while graduate students and above accounted for 20.1%. For monthly income, the gap between the proportions of various income groups was small. Among them, the highest proportion was the group with a monthly income in the range of 5,001–10,000 yuan (34.5%). The rest of the income groups were arranged in descending order: those with 10,001–20,000 yuan (27.5%), those below 5,001 yuan (26.5%), and those more than 20,000 yuan (11.5%). Those who worked as company staff accounted for the highest proportion (30.2%), followed by those who were part of the student group (22.4%).

**Table 1:** Characteristics of respondents.

Demographic variable	Category	Number of samples	Proportion (%)
Gender	Male	257	52.8
	Female	230	47.2
Age	Under 18 years old	38	7.8
	18–25 years old	156	32.0
	26–35 years old	162	33.3
	36–50 years old	69	14.2
	51–60 years old	39	8.0
	Over 60 years old	23	4.7
Education level	High school (technical secondary school) and below	90	18.5
	Junior college	122	25.1
	Undergraduate	197	36.3
	Master’s degree or above	78	20.1
Monthly income	Less than 5,001 yuan	129	26.5
	5,001–10,000 yuan	168	34.5
	10,001–20,000 yuan	134	27.5
	More than 20,000 yuan	56	11.5
Occupation	Student	109	22.4
	White Collar	147	30.2
	Blue Collar	60	12.3
	Professional and technical personnel	41	8.4
	Government officials	78	16.0
	Soldier	9	1.8
	Retirees	21	4.3
	Others	22	4.5
Annual number of trips	once	218	44.8
	twice	130	26.7
	3 times	84	17.2
	4 times or more	55	11.3
Average daily view time of short-form videos	less than 30 min	219	45.0
	30 min to 1 h	144	29.6
	more than 1 h but less than 2 h	86	17.7
	2 h or more	38	7.8
Whether there is a travel experience after watching a tourism short-form video	Yes	207	42.5
	No	280	57.5

On watching duration of short-form videos, over 70% of respondents spent less than an hour viewing short videos per day on average, of which 45% reported watching under 30 min worth of videos. Nearly 25% of respondents spent more than 1 h on short videos, including 7.8% of those determined as heavy users, spending more than 2 h a day watching short videos. This showed that short video platforms have become a daily source of both information and entertainment for the larger public.

### Reliability test and exploratory factor analysis

SPSS23.0 software was used to analyze 487 valid data collected from formal research. Cronbach’s alpha was used to test the reliability of the questionnaire, and KMO and Bartlett’s tests were used to test the content and construct validities of the scale. Results showed that Cronbach’s Alpha coefficient of the total scale is 0.921, the reliability of the overall scale is strong, and the reliability of Cronbach’s Alpha coefficient of each observed variable is greater than 0.8, indicating that the variables have good internal consistency and reliability.

Simultaneously the KMO value of the total scale was 0.944, indicating that the overall validity of the questionnaire was good. The KMO values of audience involvement, cognitive image, affective image, psychological distance, and travel intention of the subscales were at 0.934, 0.953, 0.759, 0.722, and 0.713, respectively, all of which were greater than 0.7. Bartlett’s sphericity test value was also significant (Sig. < 0.001), indicating that the validity of the questionnaire meets the premise requirements of factor analysis.

Next, the principal component analysis was used to carry out factor extraction and factor rotation carried out using the maximum variance orthogonal rotation method. In Table 2, a total of seven factors were obtained for behavioral involvement, emotional involvement, referential reflection, cognitive image, affective image, psychological distance, and travel intention. The cumulative variance explained reached 67.272%, indicating good representativeness. The factor loadings coefficients are presented in the table below, with each measured item having a factor loading greater than 0.5 and falling into its corresponding factor. Summarily, the results of the scale’s factor extraction are consistent with the dimensional divisions found in well-established scales, and the overall scale has good construct validity.

**Table 2:** The results of EFA.

Variable		Item	Factor loading	Mean	Weight (%)
Audience involvement	Behavioral involvement	AI11	0.828	4.307	11.641
		AI12	0.768		
		AI13	0.737		
		AI14	0.724		
		AI15	0.755		
		AI16	0.784		
	Emotional involvement	AI21	0.741	3.415	20.869
		AI22	0.766		
		AI23	0.747		
		AI24	0.750		
		AI25	0.749		
	Referential reflection	AI31	0.756	2.194	26.798
		AI32	0.781		
		AI33	0.763		
Cognitive image		CI1	0.713	7.95	48.285
		CI2	0.687		
		CI3	0.685		
		CI4	0.661		
		CI5	0.693		
		CI6	0.726		
		CI7	0.721		
		CI8	0.756		
		CI9	0.759		
		CI10	0.740		
		CI11	0.807		
		CI12	0.827		
		CI13	0.812		
Affective image		EI1	0.761	2.674	55.512
		EI 2	0.765		
		EI 3	0.746		
		EI 4	0.699		
Psychological Distance		PD1	−0.822	2.25	61.593
		PD2	−0.808		
		PD3	−0.821		
Travel intention		BI1	0.774	2.101	67.272
		BI2	0.763		
		BI3	0.726		

### Confirmatory factor analysis

AMOS software was used to perform the CFA. The results showed that the final CFA model has good fitting index (*χ2* = 840.024, *df* = 616, *χ2/df* = 1.364, *RMSEA* = 0.027, *GFI* = 0.918, *NFI* = 0.924, *IFI* = 0.979, *TLI* = 0.977, *CFI* = 0.978) with all the fit indicators meeting the general research criteria (Hu and Bentler, 1999). As shown in the convergent validity results in Table 3, the standardized factor loadings for each measure of each variable were all greater than 0.6, the combined reliability (CR) was all greater than 0.7, and the average variance extraction (AVE) was all greater than 0.5 showing that it met the test criteria. This indicates that the variables of audience involvement, cognitive image, affective image, psychological distance, and travel intention had good convergent validity.

**Table 3:** The results of the Confirmatory factor analysis.

Variable	Item	Factor Loading	CR	AVE
Audience involvement	behavioral involvement	0.766	0.824	0.61
	emotional involvement	0.806		
	referential reflection	0.770		
Behavioral involvement	AI11	0.869	0.907	0.621
	AI12	0.831		
	AI13	0.714		
	AI14	0.751		
	AI15	0.760		
	AI16	0.794		
Emotional involvement	AI21	0.842	0.887	0.611
	AI22	0.794		
	AI23	0.723		
	AI24	0.781		
	AI25	0.763		
Referential reflection	AI31	0.810	0.832	0.622
	AI32	0.787		
	AI33	0.769		
Psychological distance	PD1	0.812	0.829	0.617
	PD2	0.763		
	PD3	0.781		
Cognitive image	CI1	0.717	0.943	0.563
	CI2	0.724		
	CI3	0.711		
	CI4	0.674		
	CI5	0.682		
	CI6	0.722		
	CI7	0.724		
	CI8	0.764		
	CI9	0.768		
	CI10	0.759		
	CI11	0.839		
	CI12	0.847		
	CI13	0.803		
Affective image	EI1	0.784	0.832	0.556
	EI2	0.832		
	EI3	0.667		
	EI4	0.686		
Travel intention	BI1	0.784	0.827	0.615
	BI2	0.828		
	BI3	0.739		

The AVE method was used in this study to assess the differential validity. Following Table 4, the square root of the AVE values for the latent variables is located diagonally, and the correlation coefficients are in the lower left-hand part of the matrix. Results show that the standardized correlation coefficients for each variable are all less than the square root of the AVE values and the measurement results are in line with the intended objectives and the discriminant validity of this study is good (Fornell and Larcker, 1981).

**Table 4:** Correlation coefficient matrix.

	Audience involvement	Psychological distance	Cognitive image	Affective image	Travel intention
Audience involvement	**0.781**				
Psychological distance	−0.378^**^	**0.785**			
Cognitive image	0.417^**^	−0.352^**^	**0.750**		
Affective image	0.407^**^	−0.234^**^	0.466^**^	**0.746**	
Travel intention	0.420^**^	−0.283^**^	0.505^**^	0.524^**^	**0.784**

The symbol “**” shows *p* < 0.01. The bold values show the square root of the AVE values for the latent variables.

### Structural model analysis

**Figure 2:**
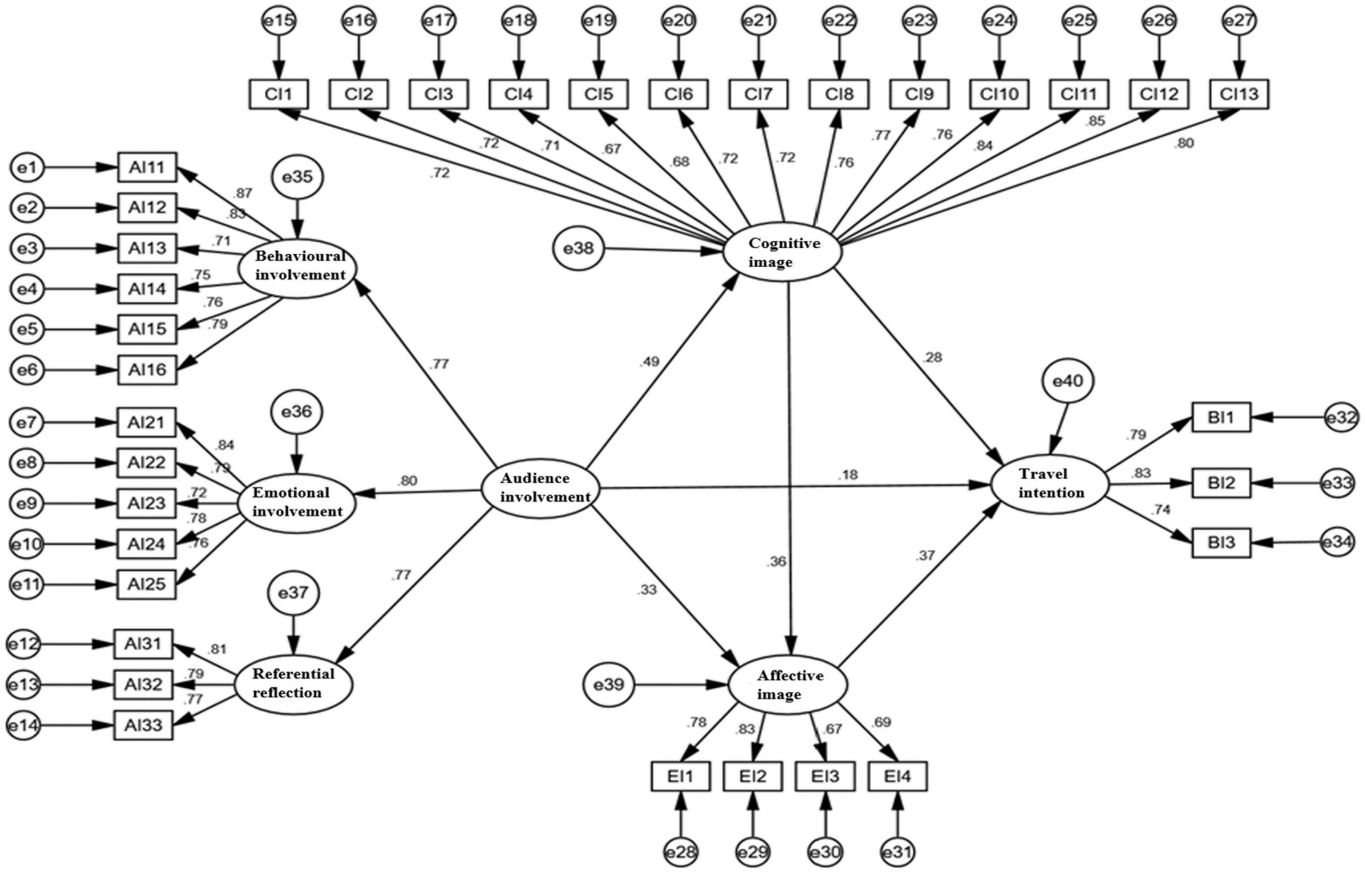
Structural equation model.

After obtaining good model fitness, Table 5 shows the results of the path coefficients for the structural equation model and the hypothesis testing (see also overview in Figure 2). There, audience involvement has a significant positive impact on travel intention with a standardized path coefficient of 0.185 and a *p* value less than 0.05, indicating that H1 is tenable. Audience involvement also has a significant positive impact on the cognitive image with a standardized path coefficient of 0.489 and a *p* value of less than 0.001, indicating H2a is tenable as well.

**Table 5:** Results of the SEM.

Hypothesis	Standard error	S.E.	C.R.	*P*	Result
H1	0.185	0.084	3.123	0.002	Accepted
H2a	0.489	0.057	8.297	***	Accepted
H2b	0.328	0.079	5.38	***	Accepted
H3	0.358	0.075	6.388	***	Accepted
H4a	0.284	0.081	5.197	***	Accepted
H4b	0.374	0.066	6.257	***	Accepted

The symbol “***” shows *p* < 0.001.

Audience involvement again has a significant positive impact on the affective image with the standardized path coefficient of 0.328 and a *p* value less than 0.001, indicating that H2b is likewise tenable. Regarding the “cognitive-affective” structure of the destination image, the results show that the cognitive image has a significant positive impact on the affective image given its standardized path coefficient of 0.358 and a *p* value of less than 0.001, indicating that H3 is tenable. Cognitive image has a significant positive impact on travel intention with the standardized path coefficient of 0.284 and *p* value of less than 0.001, indicating that H4a is also tenable. Finally, Affective image has a significant positive impact on travel intention with a standardized path coefficient of 0.284 and a *p* value of less than 0.001. This likewise indicates that H4b, following the other hypotheses mentioned is also tenable.

### Mediation effect test

The study uses the Bootstrap method to run 5,000 times in AMOS23.0 to obtain the level values of Bias-Corrected and Percentile at 95% confidence to verify the mediation effect.

The research shows that the bootstrap confidence interval does not contain 0, and the corresponding total effect, indirect effect, and direct effect do exist. The specific test results are shown in Table 6. In the total effect test, the total effect value of “audience involvement→travel intention” is at 0.512, which does not contain 0 in the lower and upper value intervals of Bias-Corrected and Percentile 95% CI and indicating that the total effect exists. In the indirect effect test, the indirect effect value of “audience involvement→cognitive image→travel intention” was at 0.139, the indirect effect value of “audience involvement→affective image→travel intention” was at 0.123, and the indirect effect value of “audience involvement→cognitive image” was at 0.123. The indirect effect value of “image→affective image→travel intention” is 0.065 and does not contain 0 in the lower and upper value intervals of Bias-Corrected and Percentile 95% CI, indicating that the indirect effects of the three exist.

**Table 6:** The results of the mediation effect test.

	Effect	Bias-corrected		Percentile	
		95%CI		95%CI	
		Lower	Upper	Lower	Upper
**Total effect**					
Audience involvement→Travel intention	0.512	0.407	0.604	0.407	0.604
**Indirect effect**					
Audience involvement→Cognitive image→Travel intention	0.139	0.088	0.209	0.082	0.202
Audience involvement→Affective image→Travel intention	0.123	0.07	0.201	0.06	0.188
Audience involvement→Cognitive image→Affective image→Travel intention	0.065	0.036	0.112	0.034	0.108
**Direct effect**					
Audience involvement→Travel intention	0.185	0.054	0.304	0.061	0.31

In the direct effect test, the direct effect value of “audience involvement→travel intention” is at 0.185, which does not contain 0 in the lower and upper value intervals of Bias-Corrected and Percentile 95% CI, indicating that the direct effect also exists simultaneously. In sum, both direct and mediating effects between the influence paths of audience involvement and travel intention exist. Cognitive image, affective image, and “cognitive-affective image” all play a part in mediating audience involvement and the travel intention effect. These results indicate that H5a, H5b, and H5c are all tenable as well.

### Moderating effect test

The relationship between audience involvement and destination image (cognitive/affective image), audience involvement, and travel intention is affected by psychological distance, with the moderating effect of psychological distance being further tested. SPSS 23.0 statistical software was used to establish a multiple regression model with interaction terms whose purpose was achieved through hierarchical regression analysis (Yin et al., 2020).

First, taking audience involvement as an independent variable, psychological distance as a moderator variable, and cognitive image as a dependent variable, a regression equation and an interaction term of audience involvement and psychological distance were both established along with the multiplication of the two variables added to the model to test whether the interaction was significant. The hierarchical regression analysis results are shown in Table 7 below.

**Table 7:** A test of the moderating role of psychological distance in the relationship of audience involvement and cognitive image.

	Cognitive image		
	Model 1	Model 2	Model 3
	*β*	*β*	*β*
Audience involvement	0.417^***^	0.331^***^	0.374^***^
Psychological distance		−0.227^***^	−0.178^***^
Audience involvement × Psychological distance			−0.139^**^
*R* ^2^	0.174	0.218	0.234
*F*	101.926^***^	67.356^***^	49.295^***^

^**^p < 0.01; ^***^p < 0.001.

Following Model 3 in Table 7, the interaction term of audience involvement and psychological distance has an evident negative impact on cognitive image, and the interaction term has a significant effect at the level of *p* < 0.05 and a *β* coefficient of −0.139 indicating that psychological distance is significantly affected by audience involvement. Psychological distance therefore negatively moderated the relationship between audience involvement and cognitive image, indicating that H7a is thereby established.

Second, taking audience involvement as an independent variable, psychological distance as a moderator variable, and affective image as a dependent variable, an interaction term of audience involvement and psychological distance was established, and the multiplied variable of the two was added to the model to test whether the interaction was significant. The hierarchical regression analysis results are shown in Table 8 below. Model 3 suggests that audience involvement and psychological distance do not have a significant negative impact on affective image (= −0.007, *p* > 0.05), indicating that psychological distance does not have a negative moderation in the impact of audience involvement on affective image. Thus, H7b is not supported.

**Table 8:** A test of the moderating role of psychological distance in the relationship of audience involvement and affective image.

	Affective image		
	Model 1	Model 2	Model 3
	*β*	*β*	*β*
Audience involvement	0.407^***^	0.371^***^	0.373^***^
Psychological distance		−0.094*	−0.092
Audience involvement × Psychological distance			−0.007
*R* ^2^	0.165	0.173	0.173
*F*	96.092^***^	50.601^***^	33.673^***^

^*^p < 0.05; ^***^p < 0.001.

Third, taking audience involvement as an independent variable, psychological distance as a moderator variable and travel intention as a dependent variable, an interaction term of audience involvement and psychological distance is established, and the multiplication of the two variables is added to the model to test whether the interaction is significant. The hierarchical regression analysis results are shown in Table 9 below.

**Table 9:** A test of the moderating role of psychological distance in the relationship of audience involvement and travel intention.

	Travel intention		
	Model 1	Model 2	Model 3
	*β*	*β*	*β*
Audience involvement	0.42^***^	0.365^***^	0.397^***^
Psychological distance		−0.145^**^	−0.108^*^
Audience involvement × Psychological distance			−0.104^*^
*R* ^2^	0.176	0.194	0.203
*F*	103.69^***^	58.275^***^	41.123^***^

^*^p < 0.05; ^**^p < 0.01; ^***^p < 0.001.

Model 3 also suggests, following Table 9, that audience involvement and psychological distance have a significant negative impact on travel intention, the interaction term has a significant effect at the level of *p* < 0.05, and the *β* coefficient is −0.104, indicating that psychological distance has a significant impact on audience involvement. Psychological distance negatively moderates the relationship between audience involvement and travel intention, thereby supporting H6.

## Discussion

### The influence of audience involvement on destination image and travel intention

Results show audience involvement has a significant positive impact on destination image and travel intention. Specifically, the higher the audience’s involvement in short-form travel videos, the more positive the evaluation of the tourism destination’s cognition and affective image. This mirrors Greenwald and Leavitt (1984) view of audience involvement in advertising where they posit that the more the audience focuses on the short-form video content, the more destination tourism element symbols can be received, and the better the evaluation generated based on the narrative content, ultimately forming positive feedback on the cognitive image of the destination. Affected by the positive emotional infection or word-of-mouth recommendation of the publisher, the audience also has emotional involvement and reference reflection on their travel experience, has a good impression of the destination image, and finally forms a positive affective image (Long et al., 2021). From the two-dimensional structure of “cognition-emotion” of destination image, compared with affective image (*β* = 0.489), audience involvement has a greater impact on cognitive image (*β* = 0.328) which is consistent with previous research results (Fu et al., 2016).

From the path of “audience involvement→travel intention,” the higher the audience’s involvement in short-form travel videos, the stronger the travel intention is to the destination where said video was recorded. The strong social interaction attributes and atmosphere creation of short videos are conducive for audiences to have a deeper emotional involvement and empathy, immerse themselves audience into the story, induce their fantasy about the video shooting content or themselves, and enhance the emotional experience of the video shooting location. This experience further fuels the idea of tourists wanting to experience actual tourist destinations. Some scholars mentioned that Internet Word of Mouth (IWoM) has different effects on tourists’ destination decisions (Xu and Yao, 2020). Currently, most short-form travel videos are shot by tourists based on their own travel experiences. To record the journey, share experiences, or for purposes of vanity, they tend to upload their short videos to social media platforms hoping that they will be released through the attention and recognition of others. Gaining a strong sense of self-satisfaction also promotes potential tourists’ interest in tourist destinations (Zeng and Li, 2019). Meanwhile, identifying and liking the publisher or the content of the program will also arouse the audience’s intention to visit the shooting location (Yi, 2020). Therefore, audience involvement has a significant positive impact on travel intention.

### The influence of destination image on travel intention

Destination cognitive image and affective image both have significant positive effects on travel intention. Scholars have proposed that tourists’ evaluation of destination image will ultimately affect their tourism behaviors (Chaulagain et al., 2019), which this study’s results also support. When potential tourists have not traveled to a certain place, the destination image is regarded as a true portrayal of the destination and has a key impact on tourists’ decision to travel and their subsequent travel plans (Chen and Tsai, 2007). Therefore, when tourists have a positive evaluation of the cognitive and affective image of a destination, it influences and promotes tourists’ willingness to go to the tourist destination. Compared with cognitive image, affective image has a greater impact on travel intention. The former has an influence coefficient of 0.28, while the latter has an influence coefficient of 0.37. This coincides with conclusions drawn from previous research (Fu et al., 2016).

### The mediating role of destination image

Destination cognitive image and affective image play a mediating role between audience involvement and travel intention, which also verifies the conclusions of previous studies. The audience involvement has a significant direct impact on travel intention and indirectly affects tourists’ travel intention through the cognitive and affective image of the destination. When the audience is more involved in the presented content, it can form positive feedback on the cognitive or affective image of the tourist destination, eventually prompting the audience to generate visit intention, recommendation willingness, and potential travel to the destination for an actual tourist experience. Specifically, this study found that cognitive image can significantly and positively affect affective image, which supports the “cognition-emotion” theory of destination image, i.e., cognition is a necessary condition for emotion generation and people always recognize what is happening around them first and then generate corresponding emotions (Lee et al., 2005). Furthermore, the study verifies the path relationship of “audience involvement→cognitive image→affective image→travel intention,” supporting the claim of the “cognitive-emotional” image chain mediation effect (Ding et al., 2019). From a standardized effect size perspective, the direct influence of audience involvement on travel intention is significantly greater than the indirect influence of any singular path. Among the indirect effects, the indirect effect value of cognitive image is the largest, followed by the affective image. Meanwhile, “cognitive-affective image” is the smallest, and the indirect effect of the three paths is greater than the direct effect of audience involvement on travel intention.

### The moderating effect of psychological distance

Psychological distance has a negative moderating effect on the influence of audience involvement on the cognitive image and the influence of audience involvement on travel intention. Following the study’s results, when tourists perceive the destination to be spatiotemporally distant, even if the audience is more engaged in the tourism short video, the cognitive image evaluation of the destination and the possibility of an actual visit will nonetheless be negatively affected because people’s psychological proximity to things affects their behavioral decisions after interpreting information (Trope et al., 2007). Psychological distance is not significant on the path of “audience involvement→affective image” and destination psychological distance has no moderating effect between audience involvement and affective image. According to results from existing studies, psychological distance research mainly affects consumers’ social cognition and has little effect on emotional experience (Dhar and Kim, 2007) which is also reflected in this study’s results. This shows that the psychological distance mainly affects the audience’s cognition of the specific tourist destination where the short video is located, rather than the evaluation on the emotional level.

## Conclusion and recommendations

### Conclusion

In the context of short-form travel videos, this study explores the impact mechanism of audience involvement on travel intention. This study is based on involvement theory and media theory, takes audience involvement and tourists’ travel intentions as independent and dependent variables, respectively, introduces the variables of destination image and psychological distance to construct a structural model and explores the relationship between these variables.

The main research conclusions are as follows: (1) Audience involvement has a significant positive effect on travel intention. Vivid travel content can also bring a better watching experience to the audience, prompt the audience to generate higher-level behavioral involvement and reference reflection, and accelerate their travel decision-making. (2) Audience involvement significantly and positively influences destination cognitive and affective image. The more focused the audience is on short-form videos, the more symbols of the destination’s tourism elements they can receive and generate an assessment based on the narrative content, ultimately generating positive feedback on the destination image. (3) Destination cognitive and affective image each have a significant positive effect on travel intention. Positive perceptions and emotional feedback about a destination will encourage viewers to increase their willingness to visit it. (4) Destination cognitive and affective image each plays a partial mediating role between audience involvement and travel intention. The more the viewer is involved in the video, the more positive the perception and emotional image of the destination will be, and the stronger the individual’s willingness to travel. (5) Psychological distance plays a negative moderating role between audience involvement and travel intention, and on audience involvement and cognitive image, but has no significant moderating effect on audience involvement and affective image. Psychological distance plays a major role in the tourist’s cognitive evaluation of the destination, rather than on an emotional level.

### Recommendations

Based on the research findings, this study offers the following recommendations to destination management organizations from the perspective of short-form video tourism marketing.

First, destination managers should enhance the viewing experience of the short-form video audience. They should take good audience experience as the basis for creating short video content and use it to enhance the scenario-based marketing of short videos, giving audiences a sense of both presence and participation. Through the theme and content of the short video, they can create scenes that users can touch or perceive, enhance the audience’s sense of immersion, and eliminate the separation between the communicator and the audience to evoke cognitive and emotional communication and recognition from them. For example, in terms of creative themes, in addition to introducing content with local characteristics, managers should also take the tourist as the starting point, fully understanding their needs and preferences, and solving the problem of unequal ideas between creators and audiences. It is also possible to leverage the power of viewers and lead them to actively participate in the production of tourism content. Viewers can provide content and relevant material for the publisher to shoot. On the one hand, the content resources and suggestions provided by viewers can broaden the creator’s thinking limitations and enrich the diversity of content, while also greatly reducing the creator’s pressure to create and update, solving the problem of the creator’s short video quality declining or the number of updates decreasing due to the exhaustion of creative inspiration. On the other hand, fan-created themes are more closely aligned with the content preferences of fan groups, which can enhance the overall immersion and viewing experience of viewers and evoke cognitive and emotional resonance. In terms of content expression, creators can attach different narrative styles such as trip descriptions, food, attractions, tips sharing, and others, with supporting features such as music and special effects to enrich the sound and visual effects of the audience. On presentation, more attention should be paid to the image quality and clarity of the short video to reflect the characteristics of the destination and enhance the quality of the short video.

Next, destination managers should strengthen the interactive attributes of short videos to shorten the psychological distance of tourists. For example, they could stimulate interactive behaviors in terms of likes, comments, sharing, and generate interest in the destination through more in-depth involvement behaviors. Simultaneously, attention should also be given to eliminating concerns and unfamiliarity in the minds of potential travelers and shortening the psychological distance between travelers and the destination in general. For example, destination marketing can introduce practical travel guides with information on attractions, accommodation, food, transportation, and pre-trip preparation, so that audiences can spend minimal time and effort to obtain more information about the destination and get a basic overview of it. Practical information such as travel guides can also be used to help potential travelers plan their itineraries, reducing the distance that travelers feel from the destination by also reducing information differences and psychological concerns.

Finally, destination managers should create a dynamic three-dimensional image of the destination. The production of a series of short videos will not both create a more dynamic and three-dimensional image of the destination and also encourage audiences to develop the habit of catching up and watching regularly, thus enhancing their adhesion. Local administrators can present their area’s unique local charm through camera work, highlight the characteristics of various tourism elements, enrich the destination image at a micro level, and create a series of three-dimensional destination symbols. The visual presentation and rich content of the material will help to raise the audience’s awareness of the destination’s image to an entirely new level.

## Limitations and recommendations for future research

This study has some limitations. First, the influence path in this study may be influenced by other factors, that is, there is the possibility of other mediating variables. Because of the limitations of the model, other relevant variables have not been deeply explored in this study. Second, this study overlooks the effects of negative content in short videos, which has created certain research limitations. Third, this study was conducted in China and the existence of the same results in other countries needs to be further explored, while the convenience sampling method makes the data lack some generalizability and representativeness. Fourth, due to the impact of COVID-19 during the study period, the researcher mainly collected data through online questionnaires distributed on social media platforms, hence the collected questionnaires may not effectively reflect the true feelings of the respondents and may affect the accuracy of the sample data to a certain extent.

Based on the abovementioned limitations, future research can continue to explore the following aspects in depth. Subsequent research can further explore the mechanism behind the influence of the audience’s viewing experience of short videos on travel intention. A more detailed and comprehensive sorting of the influencing factors and consideration of the influence of other factors on the path (e.g., potential travel experience, perceived value, and others.) can be carried out to enrich the study. Next, future research could discuss other types of short videos, such as the impact of negative short-form travel videos. Third, in the future, consideration could be given to studying the differences between countries with different cultural backgrounds in this field, such as the differences between Eastern and Western countries, or choosing a more reasonable sampling method for the study. Finally, short-form videos could be shown to respondents during the research to ensure that the data collected is more accurate and representative, reducing bias in the findings. A typical tourist destination can be chosen as an example for field research to verify the external validity of the study’s findings.

## Data availability statement

The raw data supporting the conclusions of this article will be made available by the authors, without undue reservation.

## Author contributions

JH and GZ contributed to the conception of the study, and contributed significantly to analysis and manuscript preparation. JH collected and organized the data. GZ and SX performed the data analyses and wrote the manuscript. RL and MZ helped perform the analysis with constructive discussions. MZ is responsible for the overall project. All authors contributed to the article and approved the submitted version.

## Conflict of interest

The authors declare that the research was conducted in the absence of any commercial or financial relationships that could be construed as a potential conflict of interest.

## Publisher’s note

All claims expressed in this article are solely those of the authors and do not necessarily represent those of their affiliated organizations, or those of the publisher, the editors and the reviewers. Any product that may be evaluated in this article, or claim that may be made by its manufacturer, is not guaranteed or endorsed by the publisher.

## Footnotes

^1^ https://www.wjx.cn/
